# Sex differences in the association between plasma polyunsaturated fatty acids levels and moderate-to-severe plaque psoriasis severity: a cross-sectional and longitudinal study

**DOI:** 10.1186/s12967-023-04726-y

**Published:** 2023-11-20

**Authors:** Xin Wang, Rui Ma, Rongcan Shi, Hui Qin, Wenjuan Chen, Zengyang Yu, Yangfeng Ding, Chen Peng, Yuling Shi

**Affiliations:** 1 Department of Dermatology, Shanghai Skin Disease Hospital, Tongji University School of Medicine, 1278 Baode Road, Jing’an District, Shanghai, 200443 China; 2 Institute of Psoriasis, Tongji University School of Medicine, Shanghai, China; 3 Shanghai Skin Disease Clinical College, Fifth Clinical Medical College, Anhui Medical University, Shanghai, China; 4 Department of Dermatology, Shanghai Tenth People’s Hospital, Tongji University School of Medicine, Shanghai, China

**Keywords:** Psoriasis, Severity, Polyunsaturated fatty acids, Sex differences

## Abstract

**Background:**

Psoriasis is a chronic inflammatory skin disease with metabolic abnormalities serving as important contributors for pathogenesis and progression. Polyunsaturated fatty acids (PUFAs) have been found to be associated with human diseases, including psoriasis. However, differences and controversies exist regarding their content and roles.

**Methods:**

Plasma PUFAs concentrations were measured in 296 patients with moderate-to-severe plaque psoriasis from the Shanghai Psoriasis Effectiveness Evaluation CoHort. Disease severity was assessed using Clinician-Reported Outcomes (ClinROs), including Psoriasis Area and Severity Index (PASI), Body Surface Area (BSA) and Physician Global Assessment (PGA), as well as Patient-Reported Outcomes (PROs), including Patient Global Assessment (PtGA) and Dermatology Life Quality Index (DLQI). Multivariate generalized linear regression models (GLMs), subgroup and interaction analysis, and restricted cubic spline were used to estimate the cross-sectional associations between PUFAs concentrations and disease severity. Longitudinal assessments of PASI scores and PASI response were conducted at a 12-week follow-up. Associations between baseline plasma PUFAs levels and prospective PASI scores or PASI response were assessed using multivariate GLMs or logistic regression models.

**Results:**

Males suffered severer psoriasis and presented lower plasma docosahexaenoic acid (DHA) and arachidonic acid (ARA) levels compared to females. Among males, plasma eicosadienoic acid (EDA) level was positively associated with PASI, BSA and PGA scores, while total Omega-3 PUFAs and/or eicosapentaenoic acid (EPA) levels exhibited non-linear associations with PASI and/or BSA scores. α-Linolenic acid (ALA) was negatively, whereas ARA was positively, associated with DLQI scores. In females, Omega-3 PUFAs, including EPA, DHA, and total Omega-3 PUFAs, showed inverse associations with PASI and BSA scores. Longitudinally, plasma total Omega-6 PUFAs were positively associated with the likelihood of achieving PASI 100 at 12 weeks in males. In females, concentrations of dohomo-γ-linolenic acid were prospectively associated with an increase in PASI scores, and DHA was associated with the likelihood of achieving PASI 75 and PASI 90 decline.

**Conclusions:**

Sex differences cross-sectionally exist in disease severity and plasma PUFAs levels. The association between PUFAs and psoriasis severity also varies cross-sectionally and longitudinally between males and females. Sex differences should be considered when studying the function and clinical application of PUFAs in psoriasis.

**Supplementary Information:**

The online version contains supplementary material available at 10.1186/s12967-023-04726-y.

## Introduction

Psoriasis is a common, chronic, immune-mediated skin disease that presents at any age [1, 2]. It occurs worldwide and affects over 6 million people in China [1, 2]. Psoriasis has been regarded as a systemic inflammatory disease, with metabolic abnormalities serving as important contributors for pathogenesis and progression [3–5]. Decades ago, researchers investigated the composition and metabolism of fatty acids (FAs) in psoriasis, revealing a close association with the disease, particularly in terms of their composition and metabolism [6–8]. However, with changes in dietary habits, the composition, metabolism, and roles of FAs in the human body will change significantly. Thus, it is meaningful to analyze the latest FAs composition and metabolism of FAs in psoriasis patients and their relationship with the disease, in order to provide reference for clinical treatment.

Polyunsaturated fatty acids (PUFAs) refer to unsaturated fatty acids with two or more carbon–carbon double bonds [9]. Omega-3 PUFAs and Omega-6 PUFAs are common PUFAs widely investigated and have been reported to be associated with many human diseases [9]. However, due to populational differences, existing reports have shown differences in the content and changes of Omega-3 and Omega-6 PUFAs in human diseases, leading to controversies over their roles and the necessity of dietary supplementation [9, 10]. Differences and controversies also appear in research on skin diseases, such as atopic dermatitis, acne, and psoriasis, mainly due to considerations of population and individual genetic differences [11]. To date, only one epidemiologic study with a small sample size (85 patients) has explored the association between FAs and psoriasis, and found that PASI scores were associated with low levels of serum docosahexaenoic acid (DHA) and Omega-3 PUFAs [12]. Therefore, it is essential to investigate the latest PUFAs status in psoriasis patients and their relationship with the disease.

In this cross-sectional and longitudinal study, we aimed to observe disease severity and determine the PUFAs profiles of psoriasis patients, as well as examine the associations between PUFAs status and the severity of psoriasis. We identified significant sex differences and conducted sex-based analysis and discussion.

## Methods

### Study design and participants

**Fig. 1:**
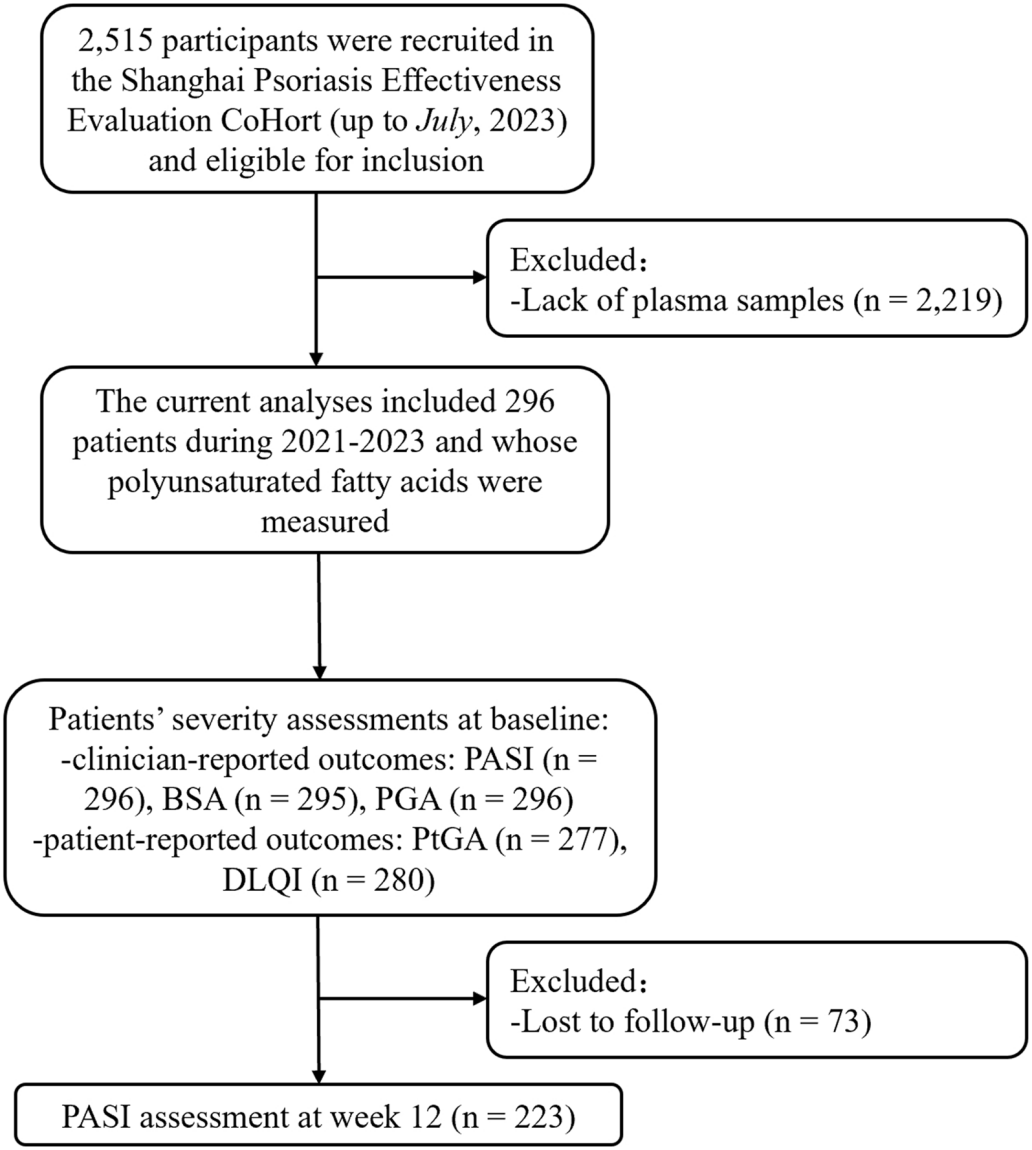
Flowchart of the participants selection. *PASI* Psoriasis Area and Severity Index, *BSA* Body Surface Area, *PGA* Physician Global Assessment, *PtGA* patient global assessment, *DLQI* Dermatology Life Quality Index

The present analysis used a cross-sectional study design and was nested in the Shanghai Psoriasis Effectiveness Evaluation CoHort (SPEECH), which was reported in our previous publication [13]. In brief, patients (≥ 18 years old) were recruited if: [1] had a diagnosis of chronic moderate-to-severe plaque psoriasis (based on Psoriasis Area and Severity Index (PASI) scores); [2] did not receive the treatment of phototherapy, conventional systemic medications (acitretin or methotrexate) in the preceding month, or biologics within the last 3 months. Among 2515 patients with moderate-to-severe plaque psoriasis recruited in the SPEECH (up to *July*, 2023), this study included 296 patients who were enrolled between 2021 and 2023 and whose PUFAs were measured. After enrollment, patients received different treatments, including phototherapy, conventional systemic medications (acitretin or methotrexate) or biological agents (adalimumab, ustekinumab, secukinumab or ixekizumab). Data, encompassing demographic information and clinical outcomes, were collected at the 12-week mark (Fig. 1) [13].

The registry (Chinese Clinical Trial Registry ChiCTR 2000036186) was performed following the principles of the Declaration of Helsinki. Ethical approvals for SPEECH project were previously described in our publication [13] and were obtained from the following institutions: Shanghai Tenth People’s Hospital (#20KT110); Ruijin Hospital (#2020821); Huashan Hospital (#KY2021-733); Changhai Hospital (#2020-27); Shanghai Jiao Tong University Affiliated Sixth People’s Hospital (#2020-KY-047); and Yueyang Hospital of Integrated Traditional Chinese and Western Medicine (#2021-129). Informed consents were obtained from all participants.

### Plasma fatty acids detection

Plasma of the venous peripheral blood was collected from the patients at baseline upon their enrollment in the SPEECH study, following a standardized protocol. Subsequently, the collected samples were centrifuged and stored at − 80 °C until analysis. The extraction and esterification of lipids were performed on 100 µL of plasma using a standard methodology with some modifications [14, 15]. Nonadecanoic acid (Sigma, USA) was used as the internal standard. Fatty acid methyl esters (FAME) (37 Component FAME Mix CDAA-252,795, ANPEL Laboratory Technologies (Shanghai) Inc.) were separated on a capillary column (30 m × 0.25 mm × 0.25 μm) (DB-wax, Agilent Technologies Inc., USA) using gas chromatography-mass spectrometry (7890B-5977B, Agilent Technologies Inc., USA). Data acquisition and processing were performed with Mass Hunter Software (Agilent Technologies Inc., USA). The proportions of PUFAs were expressed as molar proportions (mol %) of the total fatty acids. During the detection process, plasma samples were organized in batches of up to 22, which included two samples from a standard pool for quality control (QC). Coefficient of variation of QC were 4.63% for linoleic acid (LA, 18:2n6-cis), 8.01% for α-linolenic acid (ALA, 18:3n3), 9.89% for eicosadienoic acid (EDA, 20:2n6), 8.56% for dohomo-γ-linolenic acid (DGLA, 20:3n6), 10.85% for arachidonic acid (ARA, 20:4n6), 6.08% for eicosapentaenoic acid (EPA, 20:5n3) and 7.59% for DHA (22:6n3).

### Data collection and outcomes

For each patient, demographics and effectiveness outcomes including Clinician-Reported Outcomes (ClinROs) and Patient-Reported Outcomes (PROs) were collected at baseline. ClinROs included PASI (gold standard for assessing psoriasis severity, from 0 to a theoretical maximum of 72, with higher scores indicating worse disease), Physician Global Assessment [PGA, 5-point scale, from 0 (clear) to 4 (severe)] and Body Surface Area (BSA, 0–100%), and PROs included Patient Global Assessment (PtGA, 11-point scale, from 0 to 10, with higher scores indicating more severe psoriasis) and Dermatology Life Quality Index (DLQI, 30-point scale, from 0 to 30, the higher the score, the greater the impairment of quality of life) [16, 17]. After 12 weeks, PASI scores were re-assessed, and PASI 75 (defined as 75% reduction of PASI scores from baseline), PASI 90 and PASI 100 were calculated.

### Statistical analysis

The characteristics of patients were summarized using the mean with standard deviation (SD) or median with inter-quartile range (IQR) for continuous variables and number and percentage for categorical variables. The Shapiro–Wilk test was used to evaluate the normality of the data distributions. Differences between males and females were assessed using the Mann–Whitney U test for non-normally distributed data, and the chi-squared test for categorical data.

To examine the associations between plasma PUFAs levels and patients’ ClinROs or PROs scores at baseline, multivariate generalized linear regression models were performed. To determine the relationships of plasma PUFAs levels, PASI scores or PASI response at week 12, multivariate logistic regression models and multivariate generalized linear regression models were conducted. The continuous variable of PUFAs concentrations including LA, ALA, EDA, DGLA, ARA, EPA, DHA, Omega-3 PUFAs (the pool of ALA, DHA and EPA), Omega-6 PUFAs (the pool of LA, EDA, DGLA and ARA), and Omega-6/Omega-3 PUFAs ratio. Based on the literature, age, education (“high school or lower”, “college or above”), smoking history (“yes” or “no”) and alcohol using history (“yes” or “no”) were considered as potential confounders in cross-sectional study [18, 19]; while in longitudinal study, in addition to those variables mentioned above, various treatment, including acitretin, methotrexate, phototherapy, and biologics, were adjusted in statistical models [13]. Except for age, the remaining selected covariates were modeled as categorical variables. Multiple imputation was used for covariates with missing values. The number (proportion) of covariates’ missing values are shown as follows: 1 (0.34%) for Body Mass Index (BMI), 1 (0.34%) for alcohol using history, 2 (0.68%) for age, and 10 (3.38%) for education levels.

To identify the potential dose-response associations between plasma PUFAs levels and clinical scores, we used restricted cubic spline (RCS) models with four knots (5th, 35th, 65th, and 95th quantiles) to estimate *P*_overall_ and *P*_nonlinear_. Both *p*-values lower than 0.05 indicated a non-linear relationship between plasma PUFAs levels and clinical scores. β-Coefficients (β) with their corresponding 95% confidence intervals (CIs) and odd ratios (ORs) with 95% CI were used to report the change in clinical scores and PASI response, respectively. Besides, stratified by age (20–60, > 60 years old), BMI (< 23.99, 23.99–28, ≥ 28 kg/m^2^) in both sexes, and drinking history (no and yes), and alcohol using history (no and yes) in males and interaction analyses between various stratification factors and plasma PUFAs levels were performed.

Finally, we also conducted several sensitivity analyses to confirm the robustness of our major findings [1]. In our analysis, we found the non-linear relationships between PUFAs levels and disease severity in male patients. In order to better evaluate the stability and reliability of our results, we performed subgroup analysis (total males, without obese and/or overweight males); [2] Considering that there were some missing values of the covariates (e.g., age, education levels), we utilized missing indicator method to impute information and performed additional analysis [20].

All analyses were conducted using SAS 9.4 software (SAS Institute Inc., Cary, NC, USA) and R (version 4.3.1, R Development Core Team). We used the R packages “rms” for RCS analysis. The level of significance was two-sided *p*-value < 0.05.

## Results

### Patient baseline characteristics

The characteristics of the moderate-to-severe plaque psoriasis patients in this study are shown in Table 1. Of the 296 participants, there were 228 (77%) males (age 50.88 ± 15.23 years) and 68 (23%) females (age 52.00 ± 16.25 years). Notably, there were no statistically significant differences between the sexes in terms of age and the duration of psoriasis. In comparison with females, males exhibited a higher BMI, a greater proportion with a higher level of education (college or above), and a higher prevalence of smoking and drinking history.

**Table 1:** Baseline characteristics of psoriasis patients, by sex

Characteristic	Total (n = 296)	Male (n = 228)	Female (n = 68)	*p*-value
Age (years)	51.14 ± 15.45	50.88 ± 15.23	52.00 ± 16.25	0.521
Education levels				
High school or lower	164 (57.34)	121 (54.26)	43 (68.25)	0.047
College or above	122 (42.66)	102 (45.74)	20 (31.75)	
BMI (kg/m^2^)	25.23 ± 3.98	25.57 ± 3.93	24.08 ± 3.98	0.006
< 18.50	7 (2.37)	4 (1.76)	3 (4.41)	0.139
18.50–23.99	112 (37.97)	80 (35.24)	32 (47.06)	
23.99–28.00	118 (40.00)	97 (42.73)	21 (30.88)	
≥ 28.00	58 (19.66)	46 (20.26)	12 (17.65)	
Smoker, ever (yes)	169 (57.09)	163 (71.49)	6 (8.82)	< 0.0001
Alcohol use, ever (yes)	108 (36.61)	106 (46.70)	2 (2.94)	< 0.0001
Psoriasis duration (years)	15.41 ± 12.43	15.11 ± 12.78	16.35 ± 11.3	0.198

Data are shown as n (%) or mean ± SD

*P*-values are based on any differences between male and female groups examined using the Mann–Whitney U test for non-normally distributed continuous variables, chi-squared test for categorical data

### Sex differences in psoriasis severity and plasma PUFAs concentrations at baseline

As shown in Table 2, there were evident sex differences in the ClinROs and plasma PUFAs concentrations: males had higher PASI, BSA and PGA scores, whereas females had higher levels of DHA and ARA. No significant differences were observed in PROs scores between male and female patients.

**Table 2:** Psoriasis severity and plasma PUFA concentrations (mol %) by sex

Characteristic	Total (n = 296)	Male (n = 228)	Female (n = 68)	*p*-value
ClinROs				
PASI	11.5 (8.5, 15.5)	12.0 (9.3, 16.8)	9.8 (7.5, 12.0)	0.001
BSA	13.0 (9.5, 21.5)	13.6 (10.0, 24.0)	10.9 (7.5, 14.8)	< 0.0001
PGA	3.0 (2.7, 3.3)	3.9 (2.7, 3.5)	2.7 (2.3, 3.0)	0.001
PROs				
PtGA	7.0 (5.0, 8.0)	7.0 (5.0, 8.0)	7.0 (5.0, 9.0)	0.708
DLQI	8.0 (4.0, 14.0)	8.0 (4.0, 14.0)	9.0 (5.0, 14.0)	0.676
Plasma PUFAs (mol %)				
Omega-3				
α-Linolenic acid (18:3n3)	1.95 (1.44, 2.90)	1.99 (1.44, 2.99)	1.84 (1.34, 2.38)	0.206
Eicosapentaenoic acid (20:5n3)	0.69 (0.51, 1.00)	0.67 (0.50, 0.99)	0.71 (0.56, 1.05)	0.352
Docosahexaenoic acid (22:6n3)	4.42 (3.69, 5.36)	4.34 (3.53, 5.30)	4.62 (4.13, 5.72)	0.008
Omega-3 PUFAs	7.44 (6.36, 8.74)	7.34 (6.28, 8.67)	7.76 (6.47, 8.93)	0.159
Omega-6				
Linoleic acid (18:2n6-cis)	23.74 (21.98, 25.54)	23.74 (22.04, 25.56)	23.69 (21.60, 25.19)	0.343
Eicosadienoic acid (20:2n6)	0.71 (0.62, 0.79)	0.71 (0.62, 0.80)	0.70 (0.62, 0.76)	0.167
Dohomo-γ-linolenic acid (20:3n6)	0.06 (0.05, 0.08)	0.06 (0.05, 0.08)	0.06 (0.05, 0.08)	0.520
Arachidonic acid (20:4n6)	13.17 (11.03, 15.44)	12.86 (10.65, 15.20)	13.61 (11.91, 16.17)	0.032
Omega-6 PUFAs	38.21 (35.39, 40.80)	37.96 (35.34, 40.80)	38.70 (35.94, 40.95)	0.379
Omega-6/3 ratio	5.01 (4.28, 6.17)	5.08 (4.28, 6.25)	4.89 (4.27, 6.09)	0.313

Data are shown as the median (Q1, Q3), and PUFAs were expressed as molar proportions (mol %) of total fatty acids. There was one missing value on PASI in males. There were 15 missing values on PtGA in males and 4 in females, and there were 12 missing values on DLQI in males and 4 in females

*BSA* Body Surface Area, *CI* confidence interval, *ClinROs* Clinician-Reported Outcomes, *DLQI* Dermatology Life Quality Index, *PASI* Psoriasis Area and Severity Index, *PGA* Physician Global Assessment, *PtGA* Patient Global Assessment, *PROs* Patient-Reported Outcomes, *PUFAs* polyunsaturated fatty acids

*P*-values are based on any differences between male and female groups examined using the Mann–Whitney U test for non-normally distributed continuous variables

### Associations of plasma PUFAs levels with psoriasis severity at baseline

In males, EDA levels were positively associated with PASI scores (β = 7.10, 95% CI 0.61, 13.60), BSA scores (β = 14.90, 95% CI 0.56, 29.24), and PGA scores (β = 0.88, 95% CI 0.26, 1.51) (Table 3). ALA levels were positively associated with DLQI scores (β = 1.20, 95% CI 0.52, 1.89), whereas ARA levels were negatively associated with DLQI scores (β = − 0.35, 95% CI − 0.58, − 0.11) (Table 3).

**Table 3:** Association between plasma PUFAs levels and psoriasis severity in males

PUFAs (mol %)	PASI (n = 228)		BSA (n = 227)		PGA (n = 228)		PtGA (n = 213)		DLQI (n = 216)	
	β (95% CI)	*p*	β (95% CI)	*p*	β (95% CI)	*p*	β (95% CI)	*p*	β (95% CI)	*p*
Omega-3										
α-Linolenic acid (18:3n3)	− 0.46 (− 1.20, 0.28)	0.220	− 0.83 (− 2.46, 0.80)	0.318	0.00 (− 0.07. 0.08)	0.917	− 0.02 (− 0.25, 0.21)	0.866	**1.20 (0.52, 1.89)**	**0.0005**
Eicosapentaenoic acid (20:5n3)	0.78 (-0.82, 2.37)	0.340	1.45 (− 2.06, 4.96)	0.418	− 0.08 (− 0.23, 0.08)	0.315	− 0.12 (− 0.62, 0.37)	0.630	− 0.67 (− 2.16, 0.82)	0.375
Docosahexaenoic acid (22:6n3)	0.48 (− 0.16, 1.13)	0.144	0.91 (− 0.52, 2.33)	0.211	− 0.00 (− 0.07, 0.06)	0.916	0.03 (− 0.17, 0.23)	0.780	− 0.11 (− 0.72, 0.50)	0.731
Omega-3 PUFAs	0.13 (− 0.29, 0.55)	0.544	0.24 (− 0.74, 1.22)	0.630	− 0.01 (− 0.05, 0.04)	0.773	− 0.00 (− 0.14, 0.13)	0.967	0.33 (− 0.09, 0.75)	0.123
Omega-6										
Linoleic acid (18:2n6-cis)	− 0.06 (− 0.37, 0.25)	0.695	− 0.34 (− 1.02, 0.35)	0.337	0.01 (− 0.02, 0.04)	0.575	− 0.00 (− 0.10, 0.09)	0.953	0.25 (− 0.05, 0.54)	0.097
Eicosadienoic acid (20:2n6)	**7.10 (0.61, 13.60)**	**0.032**	**14.90 (0.56, 29.24)**	**0.042**	**0.88 (0.26, 1.51)**	**0.0058**	1.34 (− 0.67, 3.35)	0.190	1.08 (− 5.13, 7.28)	0.734
Dohomo-γ-linolenic acid (20:3n6)	36.96 (− 6.15, 80.06)	0.093	71.81 (− 23.26, 166.88)	0.139	1.91 (− 2.31, 6.13)	0.375	− 2.50 (− 15.68, 10.67)	0.710	3.73 (− 37.16, 44.63)	0.858
Arachidonic acid (20:4n6)	0.12 (− 0.13, 0.36)	0.360	0.08 (− 0.47, 0.63)	0.773	0.00 (− 0.02, 0.03)	0.771	− 0.01 (− 0.09, 0.07)	0.792	**− 0.35 (− 0.58, − 0.11)**	**0.0039**
Omega-6 PUFAs	0.07 (− 0.15, 0.29)	0.536	− 0.09 (− 0.58, 0.40)	0.726	0.01 (− 0.01, 0.03)	0.448	− 0.01 (− 0.08, 0.06)	0.814	− 0.14 (− 0.35, 0.07)	0.193
Omega-6/3 ratio	0.12 (− 0.32, 0.56)	0.597	− 0.00 (− 1.30, 1.29)	0.997	0.02 (− 0.03, 0.08)	0.421	0.02 (− 0.17, 0.21)	0.836	− 0.49 (− 1.07, 0.09)	0.095

Data in bold significant

PUFAs were expressed as molar proportions (mol %) of total fatty acids. The regression coefficients were adjusted for age, education (high school or lower, college or above), smoking history, and alcohol use history

*β* β-coefficient, *BSA* Body Surface Area, *CI* confidence interval, *DLQI* Dermatology Life Quality Index, *PASI* Psoriasis Area and Severity Index, *PGA* Physician Global Assessment, *PtGA* Patient Global Assessment, *PUFAs* polyunsaturated fatty acids

In females, no significant relationship was found between Omega-6 PUFAs levels and the severity scores of psoriasis patients (Table 4). Among Omega-3 PUFAs, EPA, DHA, and total Omega-3 PUFAs levels presented negative associations with PASI scores (β = − 2.67, 95% CI − 5.21, − 0.14; β = − 0.92, 95% CI − 1.72, − 0.12; β = − 0.65, 95% CI − 1.28, − 0.02), and BSA scores (β = − 5.84, 95% CI − 10.86, − 0.81; β = − 2.02, 95% CI − 3.61, − 0.44; β = − 1.42, 95% CI − 2.67, − 0.17), respectively (Table 4).

**Table 4:** Association between plasma PUFAs and psoriasis severity in females

PUFAs (mol %)	PASI (n = 68)		BSA (n = 68)		PGA (n = 68)		PtGA (n = 64)		DLQI (n = 64)	
	β (95% CI)	*p*	β (95% CI)	*p*	β (95% CI)	*p*	β (95% CI)	*p*	β (95% CI)	*p*
Omega-3										
α-Linolenic acid (18:3n3)	0.31 (− 0.95, 1.57)	0.635	0.65 (− 1.86, 3.16)	0.610	− 0.01 (− 0.19, 0.16)	0.883	0.10 (− 0.45, 0.66)	0.717	− 0.36 (− 2.07, 1.34)	0.675
Eicosapentaenoic acid (20:5n3)	**− 2.67 (− 5.21, − 0.14)**	**0.039**	**− 5.84 (− 10.86, − 0.81)**	**0.023**	0.27 (− 0.09, 0.63)	0.144	− 0.99 (− 2.08, 0.10)	0.074	− 2.91 (− 6.26, 0.43)	0.088
Docosahexaenoic acid (22:6n3)	**− 0.92 (− 1.72, − 0.12)**	**0.024**	**− 2.02 (− 3.61, − 0.44)**	**0.011**	0.04 (− 0.07, 0.16)	0.449	− 0.19 (− 0.55, 0.17)	0.297	− 0.85 (− 1.94, 0.24)	0.127
Omega-3 PUFAs	**− 0.65 (− 1.28, − 0.02)**	**0.045**	**− 1.42 (− 2.67, − 0.17)**	**0.023**	0.04 (− 0.05, 0.13)	0.383	− 0.15 (− 0.43, 0.13)	0.288	− 0.78 (− 1.61, 0.06)	0.068
Omega-6										
Linoleic acid (18:2n6-cis)	0.27 (− 0.18, 0.72)	0.243	0.84 (− 0.05, 1.73)	0.063	− 0.02 (− 0.08, 0.05)	0.581	0.12 (− 0.08, 0.32)	0.246	− 0.24 (− 0.86, 0.38)	0.447
Eicosadienoic acid (20:2n6)	5.41 (− 4.84, 15.66)	0.301	6.37 (− 14.20, 26.95)	0.544	1.02 (− 0.41, 2.45)	0.161	3.06 (− 1.37, 7.50)	0.176	1.67 (− 12.08, 15.42)	0.812
Dohomo-γ-linolenic acid (20:3n6)	− 12.40 (− 62.94, 38.14)	0.631	− 29.57 (− 130.28, 71.13)	0.565	4.17 (− 2.85, 11.19)	0.245	− 4.19 (− 26.40, 18.02)	0.711	27.32 (− 40.68, 95.32)	0.431
Arachidonic acid (20:4n6)	− 0.09 (− 0.50, 0.31)	0.644	− 0.26 (− 1.06, 0.54)	0.523	− 0.03 (− 0.08, 0.03)	0.382	− 0.05 (− 0.22, 0.13)	0.598	− 0.27 (− 0.79, 0.26)	0.318
Omega-6 PUFAs	0.08 (− 0.25, 0.41)	0.628	0.27 (− 0.39, 0.92)	0.424	− 0.02 (− 0.07, 0.02)	0.286	0.03 (− 0.11, 0.18)	0.672	− 0.32 (− 0.76, 0.13)	0.163
Omega-6/3 ratio	0.76 (− 0.14, 1.67)	0.082	**1.80 (0.01, 3.59)**	**0.049**	− 0.08 (− 0.20, 0.05)	0.244	0.25 (− 0.14, 0.65)	0.210	0.86 (− 0.36, 2.07)	0.166

Data in bold significant

PUFAs were expressed as molar proportions (mol %) of total fatty acids. The regression coefficients were adjusted for age, education (high school or lower, college or above), smoking history, and alcohol use history

*β* β-coefficient, *BSA* Body Surface Area, *CI* confidence interval, *DLQI* Dermatology Life Quality Index, *PASI* Psoriasis Area and Severity Index, *PGA* Physician Global Assessment, *PtGA* Patient Global Assessment, *PUFAs* polyunsaturated fatty acids

To further study the roles of potential confounders in the associations of plasma PUFAs levels with the severity of psoriasis, we stratified male or female patients into subgroups based on age and BMI. Additionally, we also stratified males based on smoking history and alcohol use. In the subgroups analysis of males, ALA was negatively associated with PASI scores in the 20–60 years age group (β = − 2.34, 95% CI − 3.94, − 0.73, *p*-interaction < 0.05), and EDA was positively associated with PASI scores in individuals with a BMI ranging from 23.99 to 28 kg/m^2^ (β = 11.97, 95% CI 1.86, 22.07), and with a history of alcohol use (β = 11.42, 95% CI 6.11, 22.73) (Additional file 1: Tables S1, S2). DGLA showed significantly positive association with PASI scores in males with smoking history (β = 63.73, 95% CI 10.18, 117.28) (Additional file 1: Table S2). In the subgroup analysis of females with a BMI lower than 23.99 kg/m^2^, a negative association was found between EPA and PASI scores (β = − 2.59, 95% CI − 5.15, − 0.03), while a positive relationship was observed with LA (β = 0.55, 95% CI 0.05, 1.05) and the Omega-6/3 ratio (β = 1.00, 95% CI 0.18, 1.81) (Additional file 1: Tables S3, S4).

### Dose-response associations between Omega-3 PUFAs levels and clinical scores at baseline

**Fig. 2:**
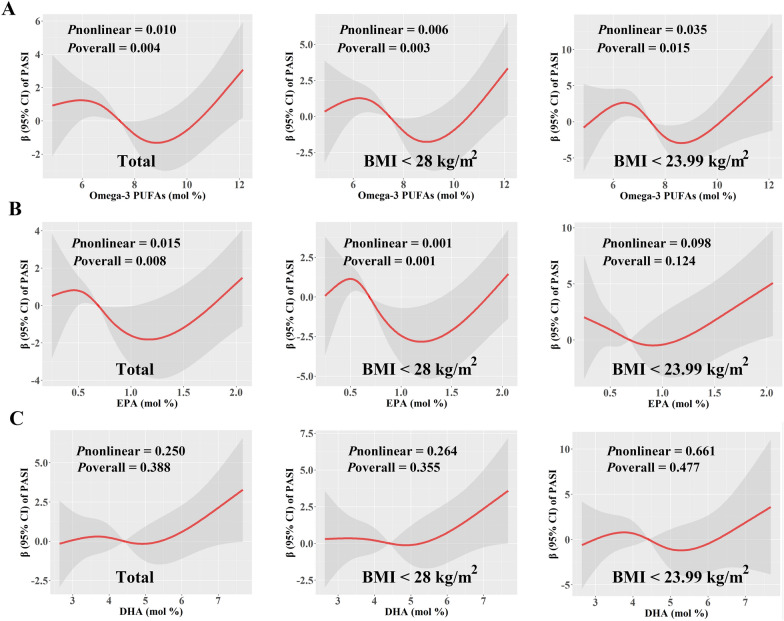
Predicted spline curves for the associations between the levels of total Omega-3 PUFAs, EPA and DHA (mol %) and PASI scores among male patients using RCS regression models. **A** Total Omega-3 PUFAs, **B** EPA, and **C** DHA in total males, males with BMI < 28 kg/m^2^, and males with BMI < 23.99 kg/m^2^, respectively. In the models, covariates including age, education, smoking history, and alcohol use history were adjusted. *β* β-Coefficient, *BMI* body mass index, *CI* confidence interval, *DHA* docosahexaenoic acid, *EPA* eicosapentaenoic acid, *PASI* Psoriasis Area and Severity Index, *PUFAs* polyunsaturated fatty acids

**Fig. 3:**
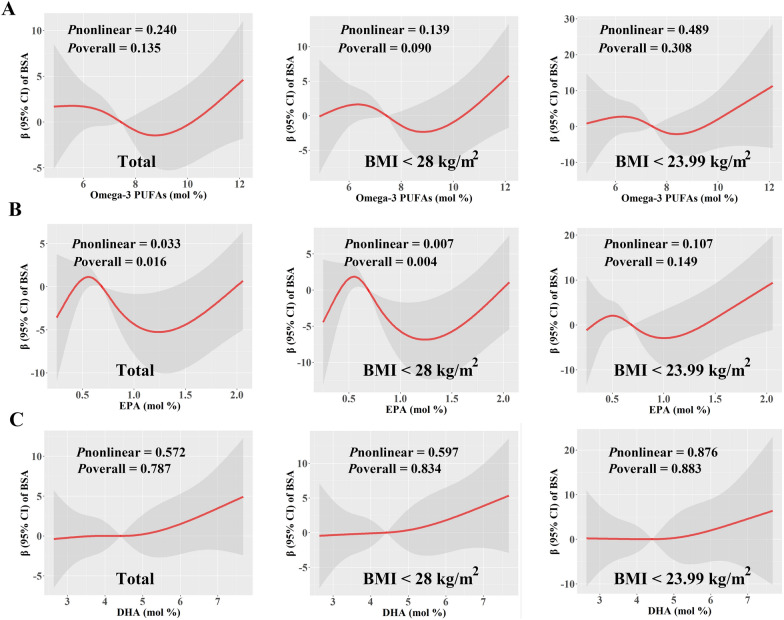
Predicted spline curves for the associations between the levels of total Omega-3 PUFAs, EPA and DHA (mol %) and BSA scores among male patients using RCS regression models. **A** Total Omega-3 PUFAs, **B** EPA, and **C** DHA in total males, males with BMI < 28 kg/m^2^, and males with BMI < 23.99 kg/m^2^, respectively. In the models, covariates including age, education, smoking history, and alcohol use history were adjusted. *β* β-Coefficient, *BMI* body mass index, *BSA* Body Surface Area, *CI* confidence interval, *DHA* docosahexaenoic acid, *EPA* eicosapentaenoic acid, *PUFAs*, polyunsaturated fatty acids

In males, we did not find a linear association between Omega-3 PUFAs and clinical scores as expected, so we further explored whether there were non-linear relationships. After adjusting for covariates, we identified non-linear associations between PASI scores and EPA and Omega-3 PUFA levels in male patients (Fig. 2). In total males, within the range of Omega-3 PUFAs increasing from 6.03 to 8.92%, the β-coefficients (95% CI) of PASI scores decreased from 1.23 (0.10, 2.37) to − 1.31 (− 2.95, 0.32), which indicated an inverse association between the PASI scores and Omega-3 PUFAs levels within this specific range. However, both below or above this range, the association was positive, resulting in a distinctive N-shaped curve (Fig. 2A). Notably, even in subgroup analyses that excluded individuals obesity or overweight, these non-linear associations still existed (Fig. 2A). Furthermore, EPA exhibited similar non-linear relationship curves with PASI scores in males, whether they had obesity or not (Fig. 2B). In the case of BSA scores, among all men and when excluding individuals with obesity, EPA levels were also associated with BSA scores in a non-linear, N-shape pattern. However, we did not find significant relationships between total Omega-3 PUFAs and BSA scores (Fig. 3).

### Associations between plasma PUFAs levels and PASI scores/response at week 12

**Fig. 4:**
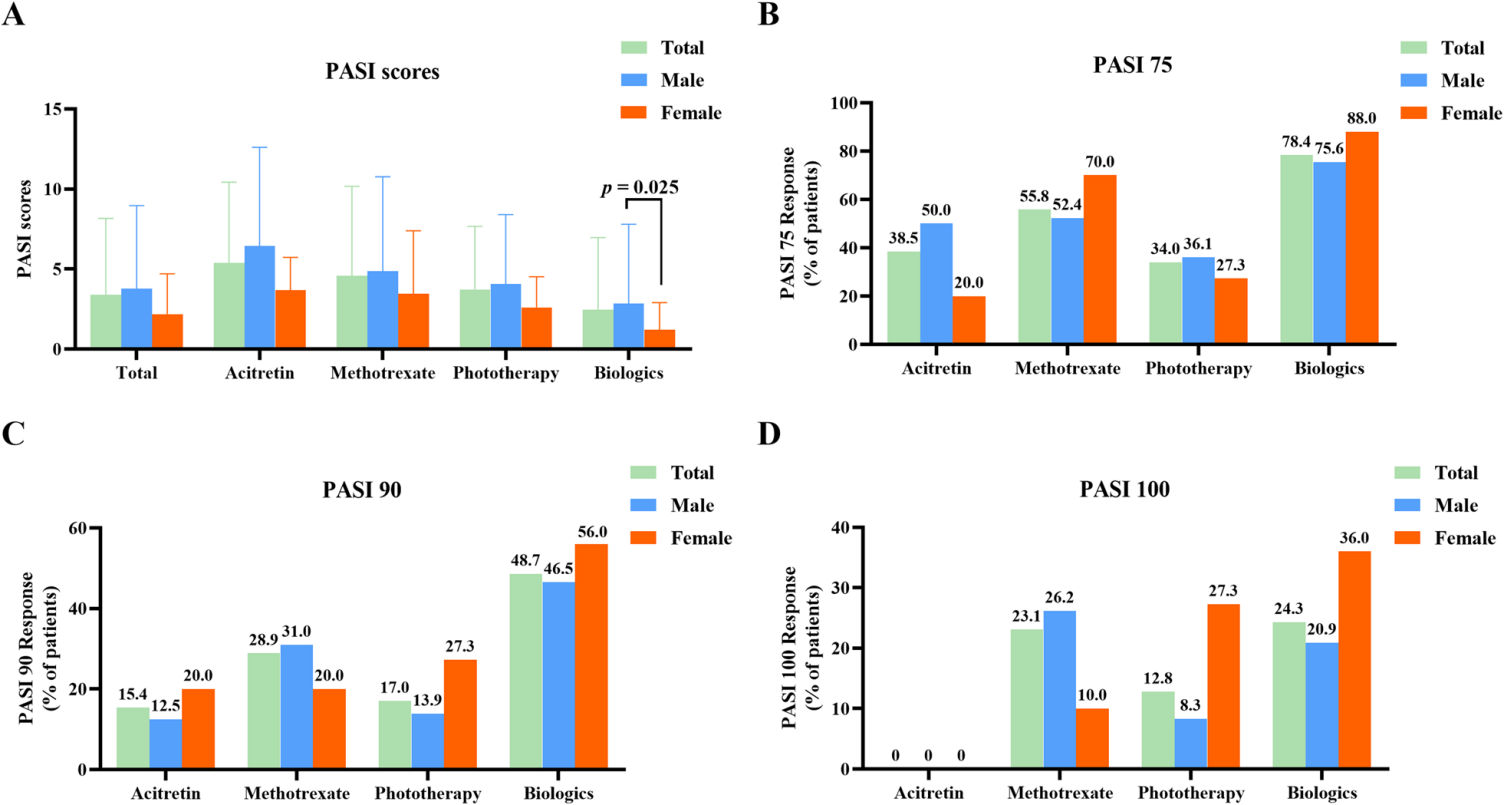
Difference of PASI scores or PASI response between male and female patients at week 12 (**A**–**D**). *PASI* Psoriasis Area and Severity Index

Initially, we did not observe any difference in achieving PASI 75, PASI 90 or PASI 100 between males and females (Fig. 4). However, compared with females, males had higher PASI scores in biologic treatment group (Fig. 4). To observe the association between plasma PUFAs levels and PASI response, we found that Omega-6 PUFAs were positively associated with the probability of achieving PASI 100 at 12 weeks (OR = 1.14, 95% CI 1.03, 1.27) in males (Table 5). In females, we found that plasma DGLA levels were positively associated with PASI scores at 12 weeks (β = 34.82, 95% CI 7.24, 62.40) (Table 6). Surprisingly, DHA was negatively associated with possibility of achieving PASI 75 or PASI 90 (OR = 0.55, 95% CI 0.31, 0.99; OR = 0.38, 95% CI 0.17, 0.84, respectively) (Table 6).

**Table 5:** Association between plasma PUFAs levels and PASI scores or PASI response at 12 weeks in males

PUFAs (mol %)	PASI scores		PASI 75		PASI 90		PASI 100	
	β (95% CI)	*p*	OR (95% CI)	*p*	OR (95% CI)	*p*	OR (95% CI)	*p*
Omega-3								
α-Linolenic acid (18:3n3)	− 0.18 (− 0.82, 0.46)	0.580	1.03 (0.79, 1.35)	0.840	0.98 (0.75, 1.30)	0.910	0.90 (0.64, 1.27)	0.545
Eicosapentaenoic acid (20:5n3)	0.14 (− 1.26, 1.54)	0.840	1.10 (0.60, 2.01)	0.767	1.55 (0.85, 2.84)	0.157	1.20 (0.63, 2.28)	0.586
Docosahexaenoic acid (22:6n3)	0.23 (− 0.35, 0.81)	0.437	1.01 (0.79, 1.30)	0.908	1.05 (0.83, 1.34)	0.681	1.25 (0.95, 1.64)	0.117
Omega-3 PUFAs	0.05 (− 0.34, 0.43)	0.810	1.02 (0.87, 1.20)	0.781	1.05 (0.90, 1.23)	0.541	1.08 (0.90, 1.30)	0.391
Omega-6								
Linoleic acid (18:2n6-cis)	− 0.07 (− 0.35, 0.20)	0.597	1.01 (0.90, 1.14)	0.867	1.04 (0.92, 1.17)	0.548	1.10 (0.95, 1.27)	0.209
Eicosadienoic acid (20:2n6)	− 1.10 (− 7.09, 4.88)	0.718	6.94 (0.50, 97.12)	0.150	2.04 (0.17, 24.93)	0.578	1.70 (0.10, 30.50)	0.717
Dohomo-γ-linolenic acid (20:3n6)	27.88 (− 11.57, 67.33)	0.166	0.02 (0.00, 358272.69)	0.645	0.00 (0.00, 64155.96)	0.452	0.00 (0.00, 2955.26)	0.202
Arachidonic acid (20:4n6)	0.10 (− 0.12, 0.33)	0.365	0.99 (0.90, 1.08)	0.815	1.03 (0.94, 1.14)	0.504	1.10 (0.99, 1.23)	0.089
Omega-6 PUFAs	0.04 (− 0.16, 0.24)	0.676	1.00 (0.92, 1.08)	0.963	1.05 (0.96, 1.14)	0.287	**1.14 (1.03, 1.27)**	**0.014**
Omega-6/3 ratio	0.12 (− 0.62, 0.38)	0.634	0.99 (0.81, 1.22)	0.932	1.07 (0.87, 1.32)	0.523	0.99 (0.77, 1.26)	0.908

Data in bold significant

PUFAs were expressed as molar proportions (mol %) of total fatty acids. The multivariate linear and logistic regression models were adjusted for age, education (high school or lower, college or above), smoking history, alcohol use history, and treatment (acitretin, methotrexate, phototherapy, and biologics)

*β* β-coefficient, *CI* confidence interval, *OR* odd ratio, *PASI* Psoriasis Area and Severity Index, *PUFAs* polyunsaturated fatty acids

**Table 6:** Association between plasma PUFAs levels and PASI scores or PASI response at 12 weeks in females

PUFAs (mol %)	PASI scores		PASI 75		PASI 90		PASI 100	
	β (95% CI)	*p*	OR (95% CI)	*p*	OR (95% CI)	*p*	OR (95% CI)	*p*
Omega-3								
α-Linolenic acid (18:3n3)	− 0.20 (− 1.01, 0.61)	0.627	1.76 (0.65, 4.78)	0.268	1.63 (0.73, 3.60)	0.231	1.04 (0.46, 2.34)	0.933
Eicosapentaenoic acid (20:5n3)	0.58 (− 1.37, 2.53)	0.560	0.16 (0.02, 1.41)	0.099	0.19 (0.03, 1.50)	0.116	0.47 (0.06, 3.90)	0.488
Docosahexaenoic acid (22:6n3)	0.06 (− 0.45, 0.58)	0.812	**0.55 (0.31, 0.99)**	**0.047**	**0.38 (0.17, 0.84)**	**0.018**	0.54 (0.25, 1.18)	0.121
Omega-3 PUFAs	0.02 (− 0.42, 0.45)	0.945	0.67 (0.41, 1.11)	0.119	0.66 (0.43, 1.02)	0.060	0.71 (0.43, 1.16)	0.170
Omega-6								
Linoleic acid (18:2n6-cis)	0.00 (− 0.25, 0.26)	0.991	1.04 (0.79, 1.38)	0.781	1.02 (0.81, 1.28)	0.854	0.96 (0.74, 1.23)	0.742
Eicosadienoic acid (20:2n6)	5.60 (− 0.57, 11.76)	0.076	0.01 (0.00, 11.86)	0.195	0.00 (0.00, 2.54)	0.093	0.06 (0.00, 47.05)	0.407
Dohomo-γ-Linolenic Acid (20:3n6)	**34.82 (7.24, 62.40)**	**0.013**	**0.00 (0.00, 0.00)**	**0.019**	0.00 (0.00, 5.79)	0.064	0.00 (0.00, 129217.71)	0.207
Arachidonic acid (20:4n6)	0.17 (− 0.09, 0.43)	0.196	0.74 (0.53, 1.03)	0.072	0.79 (0.61, 1.03)	0.084	0.94 (0.71, 1.23)	0.644
Omega-6 PUFAs	0.11 (− 0.09, 0.31)	0.288	0.84 (0.65, 1.09)	0.183	0.89 (0.74, 1.07)	0.219	0.94 (0.78, 1.14)	0.539
Omega-6/3 ratio	0.10 (− 0.45, 0.65)	0.723	1.16 (0.61, 2.19)	0.657	1.39 (0.83, 2.33)	0.205	1.29 (0.75, 2.23)	0.361

PUFAs were expressed as molar proportions (mol %) of total fatty acids. The multivariate linear and logistic regression models were adjusted for age, education (high school or lower, college or above), smoking history, alcohol use history, and treatment (acitretin, methotrexate, phototherapy, and biologics)

*Data in bold* significant, *β* β-coefficient, *CI* confidence interval, *OR* odd ratio, *PASI* Psoriasis Area and Severity Index, *PUFAs* polyunsaturated fatty acids

## Discussion

In this cross-sectional study, we observed that males experienced severer psoriasis and presented lower plasma DHA and ARA levels than females. Besides, we explored the associations between PUFAs and psoriasis severity based on sex. Among males, plasma EDA was associated with increased scores of ClinROs including PASI, BSA, and PGA. Moreover, total Omega-3 PUFAs and/or EPA were associated with PASI and/or BSA scores in a non-linear manner. In terms of PROs, ALA was negatively, whereas ARA was positively associated with DLQI scores. However, these relationships were not found in females. Plasma Omega-3 PUFAs, including EPA, DHA, and total Omega-3 PUFAs, were identified to be inversely associated with PASI and BSA scores in women. Longitudinally, plasma total Omega-6 PUFAs were positively associated with the possibility of achieving a PASI 100 response at 12 weeks in males. In females, concentrations of DGLA were prospectively associated with an increase in PASI scores, while DHA was associated with a decreased likelihood of achieving PASI 75 and PASI 90 responses (Additional file 1: Fig. S1).

In this study, we found that male patients had higher scores of PASI, BSA and PGA than their female counterparts. Although the prevalence of psoriasis is generally considered to be balanced between sexes [21, 22], the severity of psoriasis may be modified by sex due to the “nature” (e.g., different body structure, endocrine and metabolism), daily lifestyle (e.g., smoking and alcohol intake), and treatment expectations [23–25]. Previous studies have reported sex differences in psoriasis severity [26, 27]. These distinctions can also be observed in many other inflammatory conditions, such as asthma [28], myocarditis [29], atherosclerosis [30], inflammatory bowel disease [31], etc. For psoriasis, Hägg et al. demonstrated significant differences in psoriasis severity based on gender in a Swedish population of 5438 patients [26]. Another cross-sectional study enrolled 3023 Roman patients found that male sex was associated with severe or very severe PGA scores and PASI scores ≥ 10 [27]. These findings correspond with our results that men had more severe psoriasis than women. To figure out the explanations for these disparities, we also compared the duration of psoriasis, treatment approaches, and comorbidities between males and females (data not shown). However, we only found a higher prevalence of stroke (*p* = 0.030), and coronary artery disease (*p* = 0.039) in females. Considering the sex differences in psoriasis severity, dermatologists should treat and manage psoriasis patients with a sex perspective.

In addition to disease severity, we observed sex differences in plasma PUFAs profiles, with women exhibiting higher DHA and ARA levels compared to men. To our knowledge, none of the existing publications has reported sex differences in PUFAs levels among moderate-to-severe psoriasis patients. Previously cumulative observational studies have reported sex differences in Omega-3 PUFAs levels (e.g., EPA, DHA) among healthy adults, even after controlling dietary intake [32–36]. Recently, in a massive clinical population (n = 1,169,621) from the United States, females had higher median levels of DHA (+ 11.40%) and ARA (+ 4.34%) than males [37]. The underlying mechanism involved in mediating these differences may be sex hormones, presumably estrogens [33, 38, 39]. For example, estrogens regulate the expression of Delta-6 desaturase, a key enzyme in the metabolism of Omega-3 and Omega-6 fatty acids, and this hypothesis has been further supported by in vitro and in vivo studies [33, 40]. Besides, studies have documented that women tend to maintain healthier lifestyles and dietary habits compared to men [41, 42]. Thus, when exploring the association between PUFAs profiles and psoriasis severity, sex differences should be taken into consideration.

In our study, we do not find a significant difference in PROs between males and females. However, a recent cross-sectional study conducted in Dalian, China, reported an association between sex and the quality of life (QoL). The disparity between our two studies could be attributed to differences in study design, such as variations in sample size (228 males and 68 females in our study; 125 males and 60 females in Zhang’s study) and the distinct geographic locations where the research was conducted (Shanghai in our study; Dalian in Zhang’s study). Besides, patients from Zhang’s study had higher DLQI scores (mean ± SD: 11.74 ± 6.74) than our patients (9.37 ± 6.32), which underscores the individual differences among patients and the incommensurability between these two studies. Although males in our present study displayed higher ClinROs scores than females, they did not exhibit worse PROs. This discrepancy might be due to the fact that disease severity does not necessarily correlate with the quality of life [43].

The current literature suggests that psoriasis patients may have abnormal lipid metabolism, and supplementation with Omega-3 PUFAs hope to be beneficial for the management of psoriasis by diminishing inflammatory processes [11, 44]. Although previous evidence is sparse, our findings are supported by a cross-sectional study involving 85 patients, that which revealed an inverse correlation between total Omega-3 PUFAs levels and PASI scores (*p* = 0.048) [12]. However, our study boasts a relatively larger sample size, and we took into account sex differences in the analysis, which emphasizing the significance of our research. In females, we observed negative associations between EPA, DHA levels and ClinROs assessments (PASI and BSA). It is well established that psoriasis is a chronic inflammatory disease and anti-inflammation is vital important in the treatment of psoriasis [45]. Many studies have unveiled the various beneficial effects of Omega-3 PUFAs such as EPA and DHA on inflammatory diseases, as they are involved in the synthesis of anti-inflammatory metabolites [46]. They also show anti-inflammatory activities in inflammatory skin diseases, including psoriasis [47]. In psoriasis, in vivo and in vitro experiments have confirmed that Omega-3 PUFA metabolites influence multiple inflammatory signaling axis and cytokines on immune and epithelial cells, whereas the impact of dietary supplementation on psoriasis population is currently unclear [48–50].

Epidemiological studies have consistently reported a relationship between increased body weight and psoriasis [51], and a mendelian randomization study also provided compelling evidence indicating a positive association between BMI and psoriasis [52]. Thus, we performed RCS analysis to delve deeper into the impact of PUFAs on psoriasis severity, specifically considering patients with varying BMIs. Specifically, our study found a likely N-shape relationship between plasma Omega-3 PUFAs levels and the severity of psoriasis in all males, as well as in subgroups excluding those with obesity or overweight both. Among all males, plasma Omega-3 PUFAs levels below 6.03% or above 8.92% exhibited positive associations with psoriasis severity, suggesting that Omega-3 nutrient status and psoriasis action is intricate and dose-dependent. In addition, our study also found positive associations between EDA and ClinROs outcomes in males, which was consistent in subgroup analysis among overweight patients. Although this PUFA has been reported to have anti-inflammatory functions in other diseases [53, 54], the functional roles and underlying mechanisms of this PUFA in psoriasis remain largely unknown and need to be further investigated.

In addition, our study suggests that ALA levels were positively, whereas ARA levels were negatively, associated with DLQI scores. DLQI reflects the QoL during treatment, which is a subjective score of the patient [17, 55]. Till now, only a few studies have reported the roles of PUFAs in improving QoL in other diseases, such as inflammatory bowel disease and Alzheimer’s disease [56, 57]. While in the context of psoriasis, there isa dearth of relevant reports. The relevant mechanism is unclear and may be related to the impact of PUFAs on brain cognitive function [55, 56]. Collectively, based on our results from cross-sectional study, Omega-3 PUFAs was negatively associated with severity of psoriasis, while Omega-6 PUFAs had the opposite effects, indicating that taking fish oil supplements (rich in Omega-3 PUFAs) could be beneficial for the management of psoriasis. Nevertheless, this hypothesis still needs high-quality randomized controlled trials to support. Additionally, our longitudinal study did not yield consistent results regarding Omega-3 PUFAs, which could be attributed to the limited sample size and variations in treatment effects.

Our study possesses several notable strengths. First, we collected high-quality clinical data including ClinROs and PROs on psoriasis severity and QoL, which increased the reliability of our findings [13]. The consistent results from the three ClinROs outcomes substantially reduce the likelihood of false discoveries. Second, while previous studies predominantly focused on alterations in fatty acid composition in psoriasis patients compared to the general population, few explored the relationship between fatty acid levels and disease severity in psoriasis. Our study addresses this gap in the literature. Third, in this study, we used the molar percentage of PUFAs for statistical analysis. This statistical approach helps to minimize individual differences in the total amount of PUFAs, making our findings more robust. Fourth, we assessed the PUFAs status of psoriatic patients using plasma samples, a known ideal and noninvasive method that is easily accessible and highly sensitive for reflecting the PUFAs status in individuals [58]. Furthermore, we utilized standard detection assays with advanced equipment and rigorous quality control measures, which bolsters the reliability and generalizability of our study. Finally, this study conducted a sex-based analysis and found linear relationships in women as well as both linear and nonlinear relationships in men between PUFAs and disease severity. These data emphasize the importance of considering gender when studying the function and clinical application of PUFAs.

Nonetheless, our study does have several limitations. First, although a set of confounders were adjusted based on the literatures [18, 19], residual or unmeasured confounders could still exist, which might influence our findings. Second, it’s worth noting that all the psoriasis patients in our study were of Chinese ethnicity, and therefore, the generalizability of our findings to other populations may be limited. Thirdly, as with any cross-sectional epidemiologic study, the statistical associations observed in the present study cannot imply any causality. Finally, despite our study’s longitudinal design, we did not obtain consistent results when compared to the cross-sectional study. This discrepancy could be attributed to the limited sample size, variations in treatment effects, and the fact that plasma PUFAs levels at baseline may not be representative of the long-term PUFAs status of psoriatic patients. To gain a more comprehensive understanding, further research with more extensive data collection and a larger sample size, incorporating data at various time points, is required to explore factors that may modulate the effects of these fatty acids.

## Conclusion

In this cross-sectional study, we identified sex differences in both the disease severity and plasma PUFAs levels among psoriasis patients. Moreover, we cross-sectionally and longitudinally observed variations in the association between plasma PUFAs levels and psoriasis severity between men and women. Sex differences should be considered when studying the function and clinical application of PUFAs in psoriasis.

### Supplementary Information

**Additional file 1: Table S1.** Subgroup and interaction analysis between plasma Omega-3 PUFAs levels (mol %) and PASI scores in males. **Table S2.** Subgroup and interaction analysis between plasma Omega-6 PUFAs levels (mol %) and PASI scores in males. **Table S3.** Subgroup and interaction analysis between plasma Omega-3 PUFAs levels (mol %) and PASI scores in females. **Table S4.** Subgroup and interaction analysis between plasma Omega-6 PUFAs levels (mol %) and PASI scores in females. **Figure S1.** Study design and major results of the study.

## Data Availability

Data described in the manuscript, code book, and analytic code will be made available upon reasonable request.

## Abbreviations

ALA: α-Linolenic acid
ARA: Arachidonic acid
BMI: Body Mass Index
BSA: Body Surface Area
ClinROs: Clinician-Reported Outcomes
CIs: Confidence intervals
DGLA: Dohomo-γ-linolenic acid
DHA: Docosahexaenoic acid
DLQI: Dermatology Life Quality Index
EPA: Eicosapentaenoic acid
EDA: Eicosadienoic acid
FAME: Fatty acid methyl esters
FAs: Fatty acids
GLMs: Generalized linear regression models
IQR: Inter-quartile range
LA: Linoleic acid
OR: Odd ratio
PASI: Psoriasis Area and Severity Index
PASI 75: 75% Reduction of PASI scores from baseline
PASI 90: 90% Reduction of PASI scores from baseline
PASI 100: 100% Reduction of PASI scores from baseline
PGA: Physician Global Assessment
PROs: Patient-Reported Outcomes
PtGA: Patient Global Assessment
PUFAs: Polyunsaturated fatty acids
QC: Quality control
QoL: Quality of life
RCS: Restricted cubic spline
SD: Standard deviation
SPEECH: Shanghai Psoriasis Effectiveness Evaluation CoHort
