# Supplementary material for: Sex differences in the association between plasma polyunsaturated fatty acids levels and moderate-to-severe plaque psoriasis severity: a cross-sectional and longitudinal study
Source: J Transl Med. 2023 Nov 20;21:834. doi: 10.1186/s12967-023-04726-y (PMC10662930; doi:10.1186/s12967-023-04726-y)

Table S1. Subgroup and interaction analysis between plasma Omega-3 PUFAs levels (mol %) and PASI scores in males

| Subgroup | N | α-Linolenic acid (18:3n3) | |  | Eicosapentaenoic acid (20:5n3) | |  | Docosahexaenoic acid (22:6n3) | |  | Omega-3 PUFAs | |  | Omega-6/3 ratio | |
| --- | --- | --- | --- | --- | --- | --- | --- | --- | --- | --- | --- | --- | --- | --- | --- |
|  |  | β (95%CI) | *p*-interaction |  | β (95%CI) | *p*-interaction |  | β (95%CI) | *p*-interaction |  | β (95%CI) | *p*-interaction |  | β (95%CI) | *p*-interaction |
| Age^1^ |  |  | **0.016** |  |  | 0.340 |  |  | 0.185 |  |  | 0.887 |  |  | 0.977 |
| 20-60 | 149 | 0.04 (-0.79, 0.86) |  |  | 1.22 (-0.74, 3.18) |  |  | 0.43 (-0.33, 1.20) |  |  | 0.31 (-0.21, 0.82) |  |  | -0.02 (-0.73, 0.69) |  |
| >60 | 77 | **-2.34 (-3.94, -0.73)^**^** |  |  | 0.59 (-2.23, 3.41) |  |  | 0.85 (-0.39, 2.08) |  |  | -0.21 (-1.09, 0.66) |  |  | 0.50 (-0.59, 1.59) |  |
| BMI^2^ |  |  | 0.209 |  |  | 0.517 |  |  | 0.721 |  |  | 0.397 |  |  | 0.982 |
| <23.99 | 84 | -1.22 (-2.56, 0.12) |  |  | 2.50 (-0.43, 5.43) |  |  | 0.25 (-0.95, 1.45) |  |  | -0.21 (-1.15, 0.73) |  |  | 0.37 (-0.64, 1.38) |  |
| 23.99-28.00 | 97 | -0.07 (-1.26, 1.12) |  |  | 0.14 (-2.24, 2.53) |  |  | 0.79 (-0.15, 1.73) |  |  | 0.33 (-0.30, 0.96) |  |  | 0.06 (-0.88, 1.00) |  |
| ≥28.00 | 46 | -0.54 (-1.93, 0.86) |  |  | -0.45 (-3.91, 3.02) |  |  | 0.62 (-0.72, 1.97) |  |  | 0.02 (-0.88, 0.92) |  |  | 0.25 (-1.02, 1.51) |  |
| Smoker, ever^3^ |  |  | 0.152 |  |  | 0.166 |  |  | 0.470 |  |  | 0.100 |  |  | 0.117 |
| No | 65 | 0.44 (-0.95, 1.82) |  |  | 2.06 (-0.52, 4.65) |  |  | 0.77 (-0.43, 1.98) |  |  | 0.61 (-0.14, 1.35) |  |  | -0.44 (-1.51, 0.63) |  |
| Yes | 163 | -0.74 (-1.62, 0.14) |  |  | 0.08 (-1.92, 2.07) |  |  | 0.35 (-0.42, 1.12) |  |  | -0.10 (-0.64, 0.45) |  |  | 0.43 (-0.28, 1.14) |  |
| Alcohol use, ever^4^ |  |  | 0.128 |  |  | 0.622 |  |  | 0.942 |  |  | 0.410 |  |  | 0.456 |
| No | 121 | -1.00 (-2.12, 0.12) |  |  | 1.31 (-1.17, 3.80) |  |  | 0.50 (-0.57, 1.58) |  |  | -0.07 (-0.80, 0.66) |  |  | 0.40 (-0.57, 1.36) |  |
| Yes | 106 | 0.11 (-0.86, 1.08) |  |  | 0.23 (-1.80, 2.26) |  |  | 0.45 (-0.33, 1.23) |  |  | 0.27 (-0.27, 0.81) |  |  | 0.05 (-0.67, 0.77) |  |

^1^The regression coefficients were adjusted for education (high school or lower, college or above), smoking history, and alcohol use history; ^2^the regression coefficients were adjusted for age, education (high school or lower, college or above), smoking history, and alcohol use history; ^3^the regression coefficients were adjusted for age, education (high school or lower, college or above), and alcohol use history; ^4^the regression coefficients were adjusted for age, education (high school or lower, college or above), and smoking history. β, β-coefficient; BMI, body mass index; CI, confidence interval; PASI, Psoriasis Area and Severity Index; PUFAs, polyunsaturated fatty acids.

Table S2. Subgroup and interaction analysis between plasma Omega-6 PUFAs levels (mol %) and PASI scores in males

| Subgroup | Linoleic acid (18:2n6-cis) | |  | Eicosadienoic acid (20:2n6) | |  | Dohomo-γ-Linolenic Acid (20:3n6) | |  | Arachidonic acid (20:4n6) | |  | Omega-6 PUFAs | |
| --- | --- | --- | --- | --- | --- | --- | --- | --- | --- | --- | --- | --- | --- | --- |
|  | β (95%CI) | *p*-interaction |  | β (95%CI) | *p*-interaction |  | β (95%CI) | *p*-interaction |  | β (95%CI) | *p*-interaction |  | β (95%CI) | *p*-interaction |
| Age^1^ |  | 0.918 |  |  | 0.852 |  |  | 0.907 |  |  | 0.142 |  |  | 0.201 |
| 20-60 | -0.08 (-0.47, 0.32) |  |  | 4.06 (-4.10, 12.21) |  |  | 19.80 (-30.13, 69.72) |  |  | -0.05 (-0.34, 0.24) |  |  | -0.07 (-0.32, 0.19) |  |
| >60 | -0.05 (-0.59, 0.48) |  |  | 11.46 (-0.24, 23.16) |  |  | 66.77 (-20.26, 153.80) |  |  | 0.41 (-0.08, 0.91) |  |  | 0.31 (-0.14, 0.75) |  |
| BMI^2^ |  | 0.218 |  |  | 0.357 |  |  | 0.782 |  |  | 0.834 |  |  | 0.389 |
| <23.99 | -0.31 (-0.84, 0.23) |  |  | 3.19 (-7.95, 14.33) |  |  | 61.20 (-20.36, 142.76) |  |  | 0.16 (-0.22, 0.54) |  |  | 0.01 (-0.37, 0.39) |  |
| 23.99-28.00 | 0.09 (-0.41, 0.58) |  |  | **11.97 (1.86, 22.07)^*^** |  |  | -2.44 (-71.10, 66.21) |  |  | 0.13 (-0.32, 0.59) |  |  | 0.15 (-0.22, 0.52) |  |
| ≥28.00 | 0.07 (-0.66, 0.80) |  |  | 8.48 (-7.95, 24.91) |  |  | 77.06 (-20.59, 174.71) |  |  | 0.15 (-0.37, 0.67) |  |  | 0.20 (-0.33, 0.74) |  |
| Smoker, ever^3^ |  | 0.634 |  |  | 0.575 |  |  | 0.129 |  |  | 0.076 |  |  | 0.065 |
| No | -0.16 (-0.80, 0.47) |  |  | 7.37 (-3.93, 18.66) |  |  | -8.85 (-80.29, 62.60) |  |  | -0.18 (-0.57, 0.20) |  |  | -0.16 (-0.49, 0.16) |  |
| Yes | -0.05 (-0.42, 0.32) |  |  | 7.78 (-0.24, 15.80) |  |  | **63.73 (10.18, 117.28)^*^** |  |  | 0.28 (-0.03, 0.60) |  |  | 0.23 (-0.07, 0.53) |  |
| Alcohol use, ever^4^ |  | 0.556 |  |  | 0.065 |  |  | 0.592 |  |  | 0.053 |  |  | 0.215 |
| No | -0.22 (-0.72, 0.28) |  |  | -0.12 (-10.05, 9.82) |  |  | 27.96 (-31.25, 87.17) |  |  | 0.35 (-0.01, 0.72) |  |  | 0.18 (-0.14, 0.51) |  |
| Yes | 0.04 (-0.35, 0.43) |  |  | **14.42 (6.11, 22.73)^***^** |  |  | 50.00 (-13.96, 113.96) |  |  | -0.12 (-0.46, 0.21) |  |  | -0.06 (-0.36, 0.25) |  |

^1^The regression coefficients were adjusted for education (high school or lower, college or above), smoking history, and alcohol use history; ^2^the regression coefficients were adjusted for age, education (high school or lower, college or above), smoking history, and alcohol use history; ^3^the regression coefficients were adjusted for age, education (high school or lower, college or above), and alcohol use history; ^4^the regression coefficients were adjusted for age, education (high school or lower, college or above), and smoking history. β, β-coefficient; BMI, body mass index; CI, confidence interval; PASI, Psoriasis Area and Severity Index; PUFAs, polyunsaturated fatty acids.

Table S3. Subgroup and interaction analysis between plasma Omega-3 PUFAs levels (mol %) and PASI scores in females

| Subgroup | N | α-Linolenic acid (18:3n3) | |  | Eicosapentaenoic acid (20:5n3) | |  | Docosahexaenoic acid (22:6n3) | |  | Omega-3 PUFAs | |  | Omega-6/3 ratio | |
| --- | --- | --- | --- | --- | --- | --- | --- | --- | --- | --- | --- | --- | --- | --- | --- |
|  |  | β (95%CI) | *p*-interaction |  | β (95%CI) | *p*-interaction |  | β (95%CI) | *p*-interaction |  | β (95%CI) | *p*-interaction |  | β (95%CI) | *p*-interaction |
| Age^1^ |  |  | 0.879 |  |  | 0.571 |  |  | 0.806 |  |  | 0.751 |  |  | 0.974 |
| 20-60 | 149 | 0.30 (-1.28, 1.87) |  |  | 2.03 (-4.75, 0.68) |  |  | -0.89 (-1.82, 0.05) |  |  | -0.56 (-1.27, 0.14) |  |  | 0.68 (-0.29, 1.65) |  |
| >60 | 77 | 0.81 (-1.38, 3.01) |  |  | -5.06 (-10.61, 0.50) |  |  | -1.06 (-2.56, 0.43) |  |  | -0.72 (-2.01, 0.57) |  |  | 0.73 (-1.36, 2.82) |  |
| BMI^2^ |  |  | **0.032** |  |  | 0.957 |  |  | 0.463 |  |  | 0.606 |  |  | 0.146 |
| <23.99 | 84 | -0.90 (-2.29, 0.49) |  |  | **-2.59 (-5.15, -0.03)^*^** |  |  | -0.48 (-1.31, 0.36) |  |  | -0.55 (-1.14, 0.03) |  |  | **1.00 (0.18, 1.81)^*^** |  |
| 23.99-28.00 | 97 | 1.51 (-0.53, 3.56) |  |  | -4.23 (-9.04, 0.58) |  |  | -1.19 (-2.54, 0.15) |  |  | -0.61 (-1.82, 0.61) |  |  | 0.59 (-1.22, 2.40) |  |
| ≥28.00 | 46 | 1.50 (-2.22, 6.23) |  |  | -3.49 (-13.78, 6.80) |  |  | -2.63 (-5.87, 0.61) |  |  | -1.70 (-4.79, 1.40) |  |  | -0.33 (-5.11, 4.44) |  |

^1^The regression coefficients were adjusted for education (high school or lower, college or above), smoking history, and alcohol use history; ^2^the regression coefficients were adjusted for age, education (high school or lower, college or above), smoking history, and alcohol use history. β, β-coefficient; BMI, body mass index; CI, confidence interval; PASI, Psoriasis Area and Severity Index; PUFAs, polyunsaturated fatty acids.

Table S4. Subgroup and interaction analysis between plasma Omega-6 PUFAs levels (mol %) and PASI scores in females

| Subgroup | Linoleic acid (18:2n6-cis) | |  | Eicosadienoic acid (20:2n6) | |  | Dohomo-γ-Linolenic Acid (20:3n6) | |  | Arachidonic acid (20:4n6) | |  | Omega-6 PUFAs | |
| --- | --- | --- | --- | --- | --- | --- | --- | --- | --- | --- | --- | --- | --- | --- |
|  | β (95%CI) | *p*-interaction |  | β (95%CI) | *p*-interaction |  | β (95%CI) | *p*-interaction |  | β (95%CI) | *p*-interaction |  | β (95%CI) | *p*-interaction |
| Age^1^ |  | 0.489 |  |  | 0.686 |  |  | 0.751 |  |  | 0.391 |  |  | 0.844 |
| 20-60 | 0.16 (-0.38, 0.70) |  |  | 2.75 (-8.88, 14.38) |  |  | -13.85 (-69.35, 41.65) |  |  | 0.14 (-0.35, 0.63) |  |  | 0.16 (-0.21, 0.54) |  |
| >60 | 0.44 (-0.33, 1.20) |  |  | 12.80 (-7.38, 32.98) |  |  | -12.17 (-121.62, 97.28) |  |  | -0.49 (-1.15, 0.17) |  |  | -0.10 (-0.69, 0.49) |  |
| BMI^2^ |  | 0.325 |  |  | 0.694 |  |  | 0.984 |  |  | 0.421 |  |  | 0.107 |
| <23.99 | **0.55 (0.05, 1.05)^*^** |  |  | 6.51 (-3.91, 16.94) |  |  | -12.84 (-70.12, 44.44) |  |  | -0.00 (-0.45, 0.45) |  |  | 0.25 (-0.10, 0.60) |  |
| 23.99-28.00 | 0.12 (-0.69, 0.93) |  |  | 14.37 (-1.61, 30.36) |  |  | -26,25 (-138.71, 86.21) |  |  | -0.15 (-0.88, 0.58) |  |  | -0.02 (-0.59, 0.56) |  |
| ≥28.00 | 0.12 (-1.49, 1.74) |  |  | 0.25 (-48.82, 49.33) |  |  | -52.69 (-265.61, 160.23) |  |  | -0.89 (-2.21, 0.43) |  |  | -0.71 (-1.99, 0.58) |  |

^1^The regression coefficients were adjusted for education (high school or lower, college or above), smoking history, and alcohol use history; ^2^the regression coefficients were adjusted for age, education (high school or lower, college or above), smoking history, and alcohol use history. β, β-coefficient; BMI, body mass index; CI, confidence interval; PASI, Psoriasis Area and Severity Index; PUFAs, polyunsaturated fatty acids.

Figure S1. Study design and major results of the study


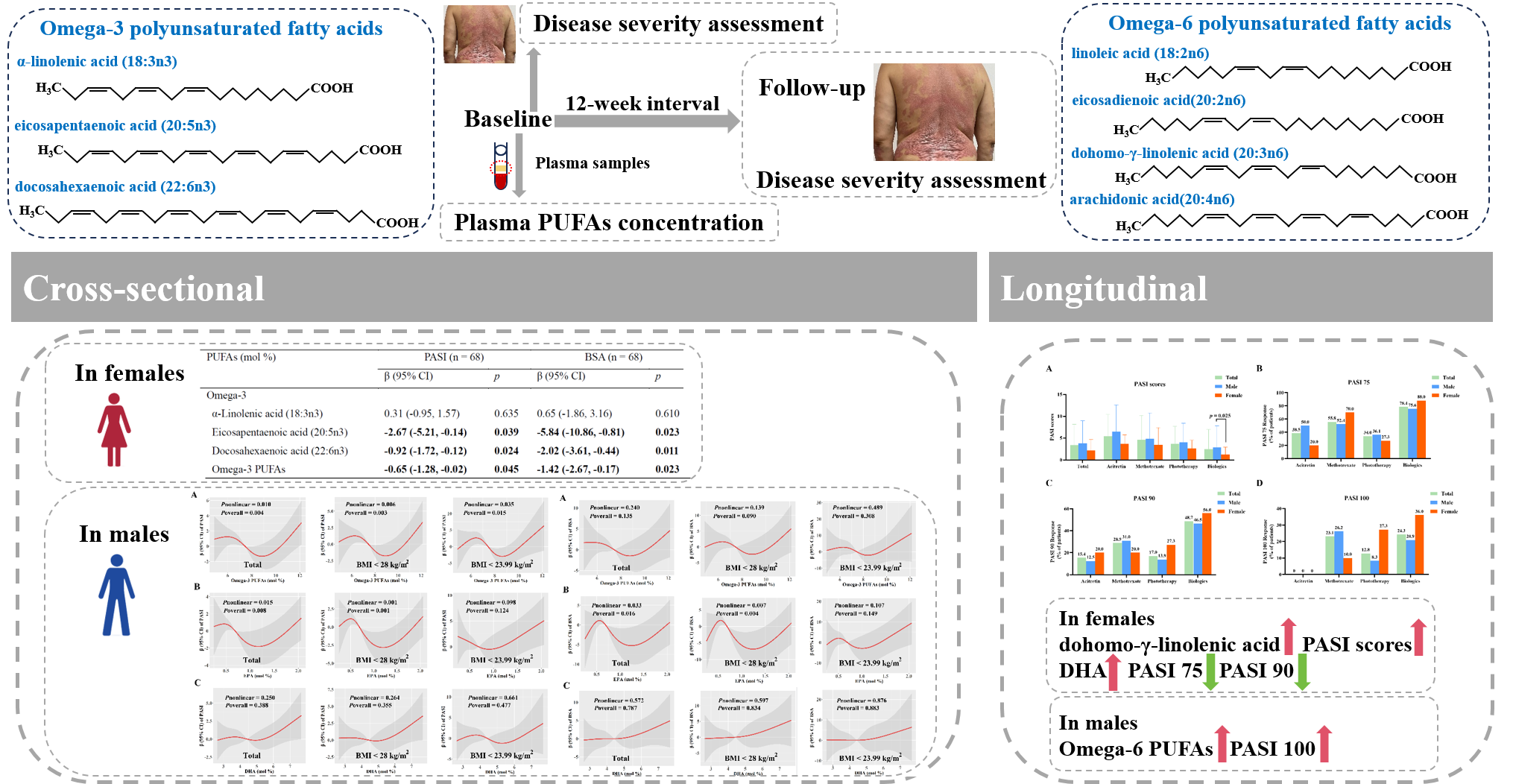

Supplement: Supplementary file 1 — Additional file 1: Table S1. Subgroup and interaction analysis between plasma Omega-3 PUFAs levels (mol %) and PASI scores in males. Table S2. Subgroup and interaction analysis between plasma Omega-6 PUFAs levels (mol %) and PASI scores in males. Table S3. Subgroup and interaction analysis between plasma Omega-3 PUFAs levels (mol %) and PASI scores in females. Table S4. Subgroup and interaction analysis between plasma Omega-6 PUFAs levels (mol %) and PASI scores in females. Figure S1. Study design and major results of the study. [file 12967_2023_4726_MOESM1_ESM.docx]
